# Closed-Form Approximations of Range Mutual Information for Integrated Sensing and Communication Systems

**DOI:** 10.3390/s26072113

**Published:** 2026-03-28

**Authors:** Zhuoyun Lai, Hao Luo, Yinlu Wang, Yue Zhang, Biao Jin

**Affiliations:** Ocean College, Jiangsu University of Science and Technology, Zhenjiang 212000, China; 231112201103@stu.just.edu.cn (Z.L.); 241112201120@stu.just.edu.cn (Y.W.); 251112201105@stu.just.edu.cn (Y.Z.); biaojin@just.edu.cn (B.J.)

**Keywords:** ISAC, range-sensing, range mutual information(RMI), range estimation

## Abstract

Sensing mutual information (SMI) is widely adopted as a performance metric for integrated sensing and communication (ISAC) to enhance both sensing and communication capabilities. However, conventional approaches derive SMI from amplitude and phase, whereas an explicit evaluation of range mutual information (RMI) remains absent. In this paper, we investigate a novel closed-form approximation of RMI for ISAC. We first derive an explicit expression for the posterior probability density function (PDF) of the target range, which is formulated as a function of the signal’s autocorrelation and cross-correlation. Furthermore, we show that under high signal-to-noise ratio (SNR), the estimated range PDF approximates a Gaussian distribution in the sensing-unconstrained scenario and a truncated Gaussian distribution in the sensing-constrained scenario. Finally, we derive closed-form approximations of the RMI in both scenarios under high SNR. In the sensing-unconstrained scenario, the RMI is proportional to the delay interval, root-mean-square bandwidth, and SNR. In the constrained scenario, we obtain a closed-form RMI approximation by introducing an entropy correction term that quantifies the impact of boundary constraints. Additionally, we employ a maximum likelihood estimation (MLE) method to assess range estimation performance. Simulation results validate the accuracy of the theoretical results and the effectiveness of the proposed approximations.

## 1. Introduction

With the evolution toward 6G wireless networks, sensing capability has emerged as an indispensable requirement, driven by the growing demand for accurate environmental information, particularly in applications such as unmanned aerial vehicle (UAV) networks [1]. Integrated sensing and communications (ISAC) has emerged as a cornerstone research area, attracting substantial interest due to its potential to simultaneously support data transmission and environmental sensing [2,3,4,5,6]. However, a fundamental challenge in ISAC systems lies in the characterization of performance metrics for both sensing and communication. Sensing metrics typically rely on indicators like the Cramér–Rao bound (CRB) [7] and minimum mean-square error (MMSE) [8], whereas communication metrics are commonly assessed via the achievable rate [9] and bit error rate [10]. The inconsistency among performance metrics has become a major obstacle to the development of ISAC systems. To tackle this issue, this work investigates the performance metrics for range sensing in ISAC systems.

### 1.1. Related Work

Recently, some research has been devoted to unifying the performance metrics for ISAC systems. Mutual information has been utilized to evaluate sensing and communication performance. For communication tasks, it corresponds to the upper bound of channel capacity. For sensing tasks, it measures the amount of useful information about the target parameters contained in the received signal. In [11], communication MI and sensing MI are jointly analyzed to characterize the sensing–communication rate region, showing that ISAC achieves higher spectral efficiency and degrees of freedom than frequency-division sensing–communication. In [12], MI is adopted as a unified metric for multi-input multi-output orthogonal frequency division multiplexing (MIMO-OFDM) ISAC systems, where a weighted sum of communication MI and sensing MI is maximized to balance the two functions. In addition, ref. [13] develops an MI-based pilot design framework for ISAC, where sensing MI and communication MI are jointly optimized to design orthogonal pilots for target detection and channel estimation. For OFDM-based ISAC waveforms, ref. [14] optimizes power allocation by maximizing radar MI under communication rate and transmit power constraints. The relationship between MI and MMSE is investigated in [15], where the MI Pareto boundary is shown to be equivalent to the MMSE Pareto boundary under optimized error weighting. In the context of waveform design and resource allocation, refs. [16,17,18] exploit MI to capture the intrinsic coupling between sensing and communication channels and to jointly optimize sensing accuracy and communication rate. Building on these MI-based formulations, characterizing MI associated with target range estimation becomes a natural next step.

However, most of the existing works mainly focus on amplitude or phase information, which has been widely adopted as a unified metric in waveform design and resource optimization problems. For example, recent waveform design studies have considered practical physical-layer constraints such as radar waveform similarity and adjustable peak-to-average power ratio (PAPR), showing that the ISAC design is still largely governed by waveform amplitude-related and power-related control variables [19]. In particular, ref. [20] proposes a zero-forcing beamforming and power allocation design for ISAC, where the dual-functional performance is adjusted through beamforming vectors and transmit power coefficients. In RIS-assisted scenarios, ref. [21] investigates transmit beamforming and RIS reflection pattern design to jointly shape communication and sensing performance, showing that the optimization variables still mainly lie in beam amplitudes and phase responses. Similarly, ref. [22] studies SNR/CRB-constrained joint beamforming and reflection design for RIS-ISAC systems, in which the communication rate and sensing accuracy are jointly controlled by transmit beamforming and RIS phase shifts. For active-RIS-enabled ISAC, ref. [23] further considers the joint optimization of the BS beamforming matrix and active RIS reflection coefficients, again indicating that the system design is essentially governed by amplitude and phase control. More recently, ref. [24] investigated hybrid beamforming and RIS phase-shift design for RIS-enabled mmWave ISAC systems, which further confirms that most existing formulations still rely on beamforming architecture and phase adjustment rather than sensing-oriented information-theoretic metrics.

Unlike conventional metrics that rely on amplitude and phase information, MI can also be extracted from the range dimension. It is essential to evaluate range-sensing performance through MI in ISAC systems. However, the explicit derivation of RMI remains unexplored, and its asymptotic closed-form approximation has yet to be established. In the absence of such analytical results, recent studies have resorted to approximations or numerical methods. Ref. [25] develops a two-stage parameter estimation framework for OTFS-based ISAC, enabling accurate estimation of delay, Doppler, range, and velocity in high-mobility scenarios. Similarly, ref. [26] shows that the delay-Doppler domain provides a natural and physically meaningful representation of wireless channels, which has become a fundamental basis for sensing and communication in high-mobility scenarios. In addition, ref. [27] showed that in near-field XL-MIMO systems, conventional angular-domain sparsity is no longer sufficient, and both angular and distance information must be jointly captured via a polar-domain representation, since the enlarged array aperture makes spherical-wave propagation effects non-negligible [28]. In multicarrier radar and ISAC scenarios, posterior relationships between the received signals and target parameters have been established to analyze the sensing information acquisition capability of the system [29]. In addition, some recent works have constructed posterior distributions based on minimum divergence principles, where range estimation performance is improved by minimizing the divergence between the true posterior and candidate distributions [30].

However, the analysis in these works is based on an idealized assumption of an infinite sensing interval, which may not hold in practical ISAC scenarios, especially when the sensing interval is limited. This paper aims to evaluate the RMI between the received signal and the target response in complex additive white Gaussian noise (CAWGN). For that purpose, we first derive an explicit expression for the posterior PDF of range between the random target response and the received signals. It is shown that posterior PDF is formulated as a function of the signal’s autocorrelation and cross-correlation. In addition, we derive closed-form approximate expressions for the RMI in high SNR in both sensing-constrained and sensing-unconstrained scenarios that can be extracted from the target response by the sensing receiver. The established relationship between RMI and traditional sensing metrics motivates the adoption of MI as a unified metric for evaluating both communication and sensing performance. To demonstrate the validity of RMI, we investigate an MLE method to evaluate range estimation performance for both sensing-constrained and sensing-unconstrained scenarios.

For clarity, the representative related works are summarized in Table 1.

### 1.2. Contributions

Based on the above observations, we study a range-sensing ISAC RMI extraction system under two scenarios: sensing-constrained scenario and sensing-unconstrained scenario. When the interval is large, the target posterior is well approximated by a Gaussian distribution. Under a finite interval, the posterior becomes a truncated Gaussian by boundary effects. Leveraging this property, we derive a closed-form approximation of RMI by explicitly modeling the posterior as a truncated Gaussian with boundary correction terms.

The main contributions of this paper are summarized as follows:

- RMI-Based ISAC system: We establish a range-sensing ISAC system model that employs RMI as a metric of range accuracy. In contrast to conventional amplitude and phase MI-based approaches, our proposed framework quantifies the RMI between the target range and the received signals with CAWGN. In addition, this design enables the system to explicitly characterize and optimize performance in the range dimension from an information-theoretic perspective.
- Posterior distribution analysis with correlation functions: To characterize the range variation of the target, we introduce a posterior PDF of the target range. Through signal correlation analysis, we establish an RMI acquisition model in which the posterior PDF is expressed explicitly in terms of the signal autocorrelation and cross-correlation functions. Our results reveal that under high SNR conditions, the PDF of the range approximately follows a Gaussian distribution in the unconstrained scenario, whereas it follows a truncated Gaussian distribution in the sensing-constrained scenario.
- Closed-form approximate expressions for RMI in high SNR: We propose novel closed-form approximations of the RMI in both scenarios. In the sensing-unconstrained scenario, the RMI is proportional to the delay interval, RMS bandwidth and SNR. In the constrained scenario, we obtain a closed-form RMI approximation by introducing an entropy correction term that quantifies the impact of boundary constraints.
- Performance evaluation of the range-sensing ISAC system: We introduce an acquisition scheme for RMI based on MLE, designed to analyze range estimation performance under both scenarios. We present numerical performance evaluations of the proposed range-sensing ISAC system under high SNR conditions. The simulation results demonstrate that the proposed approximations of RMI closely approach the theoretical results in both scenarios. Performance evaluation reveals that boundary constraints significantly reduce the RMI. These results confirm that RMI serves as an effective information-theoretic metric for quantifying the fundamental limits of range sensing in ISAC systems.

The remainder of this paper is organized as follows. Section 2 introduces the proposed range-sensing ISAC system model. Section 3 presents the derivation of the posterior distribution and the closed-form approximations of RMI in the high-SNR regime. Section 4 provides simulation results and performance analysis. Finally, Section 5 is the discussion, and Section 6 concludes the paper.

Notations: Uppercase and lowercase bold letters A and a represent a matrix and a column vector. The complex field is denoted by C. The superscripts ${\left(\cdot \right)}^{T}$, ${\left(\cdot \right)}^{*}$ and ${\left(\cdot \right)}^{H}$ denote the transpose, conjugate, and Hermitian transpose respectively. The modified Bessel function of the first kind, order zero, is denoted by $I_{0}\left(\cdot \right)$. The Euclidean norm and the magnitude are denoted by $∥\cdot ∥$ and $\left|\cdot \right|$, respectively. ℜ{·} is the real part of a complex quantity.

## 2. System Model

**Figure 1:**
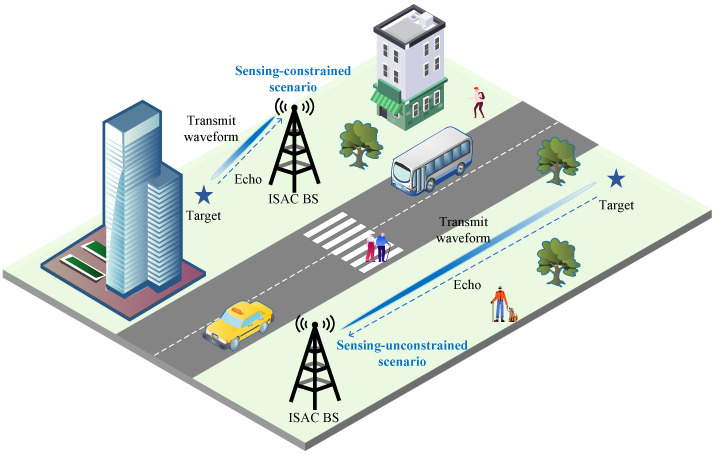
Range mutual information extraction model for ISAC in sensing-unconstrained and sensing-constrained scenarios.

As shown in Figure 1, we consider a range-sensing RMI extraction model for ISAC system. The target range is inferred from the propagation delay of the reflected echo signal. The proposed system quantifies the RMI between target range and received signals in complex additive white Gaussian noise (CAWGN). A known finite-duration baseband signal is transmitted, reflected by a single point target, and then received at the ISAC receiver. In the unconstrained scenario, the delay lies in the full observation interval. In the constrained scenario, it is limited to a finite sensing interval. The received echo signal model can be defined as

(1) $$z=\alpha e^{j\varphi }U\left(\tau \right)s+w$$

where $U\left(\tau \right)\in C^{N\times N}$ is the time delay induced by the propagation delay τ. The transmitted time-domain signal vector is $s={\left[s(0),s(1),\ldots ,s(N-1)\right]}^{T}\in C^{N\times 1}$, s(n) is the transmitted baseband signal sample at the *n*th discrete time instant, and *N* is the transmitted signal length in samples. $z={\left[z(0),z\left(1\right),\ldots ,z(N-1)\right]}^{T}\in C^{N\times 1}$ is the received vector. $w={\left[w(0),w(1),\ldots ,w(N-1)\right]}^{T}\in C^{N\times 1}$ denotes the noise vector, which is modeled as complex Gaussian noise, i.e., $w∼\mathrm{CN}\left(0,\sigma_{w}^{2}I_{N}\right)$, where $\sigma_{w}^{2}$ is the noise variance and $I_{N}$ is the N×N identity matrix.

τ denotes a normalized delay measured in sample intervals. Accordingly, the $U\left(\tau \right)s={\left[s(-\tau ),s(1-\tau ),\ldots ,s(N-1-\tau )\right]}^{T}$ represents a fractional time shift of the signal vector s. If the sampling period is $T_{s}$, the corresponding physical propagation delay and range are $t_{d}=\tau T_{s}$ and $r=cT_{s}\tau /2$, respectively, where *c* is the propagation speed. The target reflection is characterized by a constant radar cross section α and a uniformly distributed random phase φ∼U(0,2π), yielding the complex coefficient $\alpha e^{j\varphi }$.

In the theoretical analysis that follows, the closed-form RMI derivation focuses on the sensing branch of this model, namely the delayed echo observation z, its correlation with delayed replicas of the transmitted signal through U(τ), and the resulting posterior distribution of the delay parameter.

The scattering coefficient α is modeled as a Swerling 0 model, which corresponds to a non-fluctuating target model commonly adopted in radar signal processing. We assume $\alpha_{0}$ to be the arbitrary actual module of the target at a given sensing occasion. The prior distribution of α is given by

(2) $$p\left(\alpha \right)=\delta \left(\alpha -\alpha_{0}\right)$$

This modeling choice is reasonable in the considered scenario because the sensing interval is relatively short compared with the time scale of target reflectivity fluctuations. Moreover, the scattering coefficient can be treated as quasi-static within a single observation frame. The phase φ is assumed to be uniformly distributed

(3) $$p\left(\varphi \right)=\frac{1}{2\pi }$$

The sensing interval is defined in terms of the normalized discrete-time delay variable τ. Specifically, τ∈[−N/2,N/2) represents a search interval of width *N* samples centered at zero. If the sampling period is $T_{s}$, this corresponds to the physical delay interval $\left[-NT_{s}/2,NT_{s}/2\right)$ and the associated range interval $\left[-cNT_{s}/4,cNT_{s}/4\right)$. Within this interval, the delay parameter τ is assumed to be uniformly distributed as

(4) $$p\left(\tau \right)=\frac{1}{N}$$

Conditioned on (τ,α,φ), the received signal vector z is complex Gaussian with mean $\alpha e^{j\varphi }U\left(\tau \right)s$ and covariance $\sigma_{w}^{2}I_{N}$. Thus, the conditional PDF of z is given by

(5) $$p\left(z|\tau ,\alpha ,\varphi \right)=\frac{1}{{\left(\pi \sigma_{w}^{2}\right)}^{N}}\exp\left(-\frac{1}{\sigma_{w}^{2}}{∥z-\alpha e^{j\varphi }U\left(\tau \right)s∥}^{2}\right)$$

The detailed proof of (5) is provided in Appendix A.

Expanding the quadratic form in the exponent yields

(6) $$∥z-\alpha e^{j\varphi }{U\left(\tau \right)s∥}^{2}=z^{H}z-2ℜ\left\{\alpha e^{j\varphi }z^{H}U\left(\tau \right)s\right\}+{\left|\alpha \right|}^{2}s^{H}U{\left(\tau \right)}^{H}U\left(\tau \right)s$$

U(τ) in the frequency domain is defined as

(7) $$U\left(\tau \right)=F^{H}\Lambda F\;$$

where $F\in C^{N\times N}$ denotes the normalized discrete Fourier transform (DFT) matrix, $F\left(k,n\right)=1/\sqrt{N}\cdot e^{-j2\pi kn/N},\;k,n=0,1,\ldots ,N-1$. The diagonal matrix $\Lambda \in C^{N\times N}$ represents a frequency domain linear phase term, corresponding to a time-domain delay of τ. It is defined as

(8) $$\Lambda =\mathrm{diag}\left(e^{-j2\pi k\tau /N}\right),\;k=0,1,\ldots ,N-1$$

This representation enables an exact implementation of non-integer delays under a periodic discrete-time model. Since the delay parameter appears in the phase term $e^{-j2\pi k\tau /N}$, τ is dimensionless and expressed in sample units. Moreover, a unit increment of τ corresponds to one sampling interval $T_{s}$ in physical time. Since both the DFT matrix F and the diagonal phase shift matrix are unitary, and the delay operator $U\left(\tau \right)$ is also unitary, it follows that

(9) $$U^{H}\left(\tau \right)U\left(\tau \right)=I_{N}$$

According to (9), the Equation (6) can be rewritten as

(10) $$∥z-\alpha e^{j\varphi }{U\left(\tau \right)s∥}^{2}=z^{H}z-2ℜ\left\{\alpha e^{j\varphi }z^{H}U\left(\tau \right)s\right\}+{\left|\alpha \right|}^{2}E_{s}$$

where $z^{H}z$ depends only on the observation and is independent of τ,α,φ, while $E_{s}≜s^{H}s$ is the known signal energy. Accordingly, the effect of the unknown parameters is fully reflected in the correlation term $z^{H}U\left(\tau \right)s$.

The posterior PDF of τ is obtained by integrating α and φ

(11) $$p\left(\tau |z\right)=\frac{p(z,\tau )}{p(z)}=\frac{p(z,\tau )}{\int_{-N/2}^{N/2-1}p\left(z,\tau \right)d\tau }$$

Substituting (2) into it and integrating over the uniformly distributed phase φ, the posterior PDF can be rewritten as

(12) $$p\left(\tau |z\right)=\frac{\int_{0}^{2\pi }\frac{1}{2\pi }\exp\left(\frac{2\alpha_{0}}{\sigma_{\omega }^{2}}ℜ\left\{e^{j\varphi }z^{H}U\left(\tau \right)s\right\}\right)d\varphi }{\int_{-N/2}^{N/2-1}\int_{0}^{2\pi }\frac{1}{2\pi }\exp\left(\frac{2\alpha_{0}}{\sigma_{\omega }^{2}}ℜ\left\{e^{j\varphi }z^{H}U\left(\tau \right)s\right\}\right)d\varphi d\tau }$$

Using the zeroth-order modified Bessel function of the first kind $I_{0}\left(x\right)=\int_{0}^{\pi }e^{x\;\cos\;\theta }d\theta /\pi$, the above integral can be expressed as

(13) $$\int_{0}^{2\pi }\frac{1}{2\pi }\exp\left(\frac{2\alpha_{0}}{\sigma_{\omega }^{2}}ℜ\left\{e^{j\varphi }z^{H}U\left(\tau \right)s\right\}\right)d\varphi =I_{0}\left(\frac{2\alpha_{0}}{\sigma_{w}^{2}}\left|z^{H}U\left(\tau \right)s\right|\right)$$

We denote the complex correlation term as $x\left(\tau \right)=z^{H}U\left(\tau \right)s$. Since x(τ) is a complex scalar, it can be expressed in polar form $x\left(\tau \right)=\left|x\left(\tau \right)\right|e^{j\psi (\tau )}$. Then the real-part term in (13) can be written as

(14) $$ℜ\left\{e^{j\varphi }x\left(\tau \right)\right\}=ℜ\{|x\left(\tau \right)|e^{j(\varphi +\psi (\tau ))}\}=|x\left(\tau \right)|\cos(\varphi +\psi \left(\tau \right))$$

Substituting (14) into (13), the integral can be expressed as

(15) $$\int_{0}^{2\pi }\frac{1}{2\pi }\exp\left(\frac{2\alpha_{0}}{\sigma_{w}^{2}}\left|x\left(\tau \right)\right|\cos(\varphi +\psi \left(\tau \right))\right)d\varphi$$

By introducing the variable change θ=φ+ψ(τ), the phase shift can be removed and the integral becomes

(16) $$\int_{0}^{2\pi }\frac{1}{2\pi }\exp\left(\frac{2\alpha_{0}}{\sigma_{\omega }^{2}}ℜ\left\{e^{j\varphi }z^{H}U\left(\tau \right)s\right\}\right)d\varphi =\int_{0}^{2\pi }\frac{1}{2\pi }\exp\left(\frac{2\alpha_{0}}{\sigma_{w}^{2}}\left|x\left(\tau \right)\right|\cos\theta \right)d\theta$$

According to the definition of the modified Bessel function of the first kind, (13) is derived.

Therefore, the posterior PDF can be expressed as

(17) $$p\left(\tau |z\right)=\frac{I_{0}\left(\frac{2\alpha_{0}}{\sigma_{w}^{2}}\left|z^{H}U\left(\tau \right)s\right|\right)}{\int_{-N/2}^{N/2-1}I_{0}\left(\frac{2\alpha_{0}}{\sigma_{w}^{2}}\left|z^{H}U\left(\tau \right)s\right|\right)d\tau }$$

The argument of the Bessel function is determined by the magnitude of the cross-correlation between the received signal and the delay-shifted transmit signal, $|z^{H}U\left(\tau \right)s|$. This result indicates that the posterior PDF exhibits a typical single peak near the real delay. The sharpness of the distribution is jointly determined by the signal amplitude and the noise variance.

Based on the posterior PDF in (17), the RMI between the unknown τ delay parameter and the received signal z can be expressed in terms of the prior differential entropy and the posterior differential entropy as

(18) I(τ;z)=H(τ)−H(τ|z)

Since the prior distribution of τ is uniform over the observation interval [−N/2,N/2), its prior differential entropy is

(19) $$H\left(\tau \right)=\log_{2}\left(N\right)$$

Therefore, the RMI can be further written as

(20) $$I\left(\tau ;z\right)=\log_{2}\left(N\right)-E_{z}\left[\int_{-N/2}^{N/2-1}p\left(\tau |z\right)\log_{2}p\left(\tau |z\right)\;d\tau \right]$$

## 3. Range Mutual Information Analysis in Sensing-Unconstrained and Sensing-Constrained Scenarios

In ISAC systems, range estimation for the target can be performed within an unconstrained interval or a constrained range. The unconstrained scenario represents an idealized case, assuming the target delay lies within a sufficiently wide interval where boundary effects are negligible. In contrast, practical ISAC systems are often constrained by physical, operational, or algorithmic limitations, resulting in restricted effective detection ranges and thus forming constrained intervals. Therefore, in this section, we investigate the posterior PDF of the target range under both unconstrained and constrained scenarios, and further analyze the corresponding RMI performance.

### 3.1. Unconstrained Scenario

In the unconstrained scenario, the sensing interval is assumed to be sufficiently wide so that boundary effects are negligible in the high-SNR regime.

A. **Posterior PDF**

For the ISAC sensing model considered in this paper, the SNR can be characterized by the ratio $\rho^{2}≜\alpha_{0}^{2}E_{s}/\sigma_{w}^{2}$. The high-SNR regime corresponds to $\alpha_{0}^{2}E_{s}/\sigma_{w}^{2}\gg 1$, where the deterministic component of the received signal generated by the target echo is much stronger than the noise component. Moreover, the received signal exhibits a strong correlation with the real target delay, while the correlation with incorrect delay hypotheses decays rapidly.

From the posterior PDF derived in (17), it can be seen that the dependence of the posterior distribution on τ is entirely determined by the magnitude of the correlation term $\left|z^{H}U\left(\tau \right)s\right|$. Under the high SNR regime, the received signal z can be well approximated by its noise-free component as

(21) $$z\approx \alpha_{0}e^{j\varphi_{0}}U\left(\tau_{0}\right)s$$

Using the shift-invariance property of the delay operator $U^{H}\left(\tau_{0}\right)U\left(\tau \right)=U\left(\tau -\tau_{0}\right)$, $\left|z^{H}U\left(\tau \right)s\right|$ can be expressed as

(22) $$\left|z^{H}U\left(\tau \right)s\right|\approx \alpha_{0}\left|s^{H}U\left(\tau -\tau_{0}\right)s\right|$$

We now define the autocorrelation function of the transmitted signal as $R\left(\tau^{\prime }\right)≜s^{H}U\left(\tau^{\prime }\right)s$, where $\tau^{\prime }=\tau -\tau_{0}$. We define $X\left(\tau \right)≜\left|R(\tau^{\prime })\right|/E_{s}$. Then the argument of the modified Bessel function can be written as

(23) $$\frac{2\alpha_{0}}{\sigma_{w}^{2}}\left|z^{H}U\left(\tau \right)s\right|\approx 2\rho^{2}X\left(\tau \right)$$

Accordingly, the posterior PDF in (17) can be rewritten as

(24) $$p\left(\tau |z\right)\approx \frac{I_{0}\left(2\rho^{2}X\left(\tau \right)\right)}{\int_{-N/2}^{N/2-1}I_{0}\left(2\rho^{2}X\left(\tilde{\tau }\right)\right)d\tilde{\tau }}$$

B. **High-SNR approximation**

In addition, with the finite energy and bandwidth, $R(\tau^{\prime })$ can be approximated by its second-order Taylor expansion around $\tau^{\prime }=0$. Since the autocorrelation function is an even function, $R^{\prime }\left(0\right)=0$, thereby leaving only the second-order term, then

(25) $$|R\left(\tau^{\prime }\right)|\approx |R\left(0\right)|-\frac{1}{2}K{\left(\tau -\tau_{0}\right)}^{2}$$

where $\left|R\left(0\right)\right|=s^{H}s=E_{s}$ denotes the signal energy, and the curvature of the autocorrelation peak is

(26) $$K≜-{\left.\frac{d^{2}\left|R\left(\tau^{\prime }\right)\right|}{d{\left(\tau^{\prime }\right)}^{2}}\right|}_{\tau^{\prime }=0}>0$$

By evaluating the second derivative of $R(\tau^{\prime })$ and applying integration by parts, *K* can be expressed as

(27) $$K=\int {\left|\frac{ds(t)}{dt}\right|}^{2}dt=E_{s}\beta^{2}$$

$\beta^{2}$ is the root mean square (RMS) bandwidth of the transmitted signal is defined as

(28) $$\beta^{2}=\frac{\int_{-\infty }^{\infty }f^{2}{\left|S\left(f\right)\right|}^{2}df}{E_{s}}$$

and S(f) denotes the Fourier transform of the transmit signal, $E_{s}=\int_{-\infty }^{\infty }{\left|S\left(f\right)\right|}^{2}df$ is the signal energy. If the sampling period is $T_{s}$, the corresponding physical RMS bandwidth can be written as $\beta_{phys}=\beta /T_{s}$.

Substituting (27) into (25), X(τ) can be approximated as

(29) $$X\left(\tau \right)\approx 1-\frac{\beta^{2}}{2}{\left(\tau -\tau_{0}\right)}^{2}$$

The Bessel function argument in (24) becomes

(30) $$2\rho^{2}X\left(\tau \right)\approx 2\rho^{2}-\rho^{2}\beta^{2}{\left(\tau -\tau_{0}\right)}^{2}$$

Substituting (30) into (24), the posterior PDF can be rewritten as

(31) $$p\left(\tau |z\right)\approx \frac{I_{0}\left(2\rho^{2}-\rho^{2}\beta^{2}{\left(\tau -\tau_{0}\right)}^{2}\right)}{\int_{-N/2}^{N/2-1}I_{0}\left(2\rho^{2}-\rho^{2}\beta^{2}{\left(\tilde{\tau }-\tau_{0}\right)}^{2}\right)d\tilde{\tau }}$$

Equation (31) preserves the modified Bessel function structure of the posterior PDF while making the dependence of its argument on the SNR and the local curvature explicit. To further simplify this expression, we apply the standard large-argument asymptotic expansion of the modified Bessel function of the first kind

(32) $$I_{0}\left(x\right)\approx \frac{e^{x}}{\sqrt{2\pi x}}\left(1+\frac{1}{8x}+O\left(\frac{1}{x^{2}}\right)\right)$$

In the dominant neighborhood of the posterior peak, the argument $x=2\rho^{2}-\rho^{2}\beta^{2}{\left(\tau -\tau_{0}\right)}^{2}$ is of order $O(\rho^{2})$. Hence, the non-exponential terms in (32) only introduce $O(1/\rho^{2})$-order corrections and do not affect the leading Gaussian structure of the posterior PDF. Substituting *x* into (32), we obtain

(33) $$I_{0}\left(2\rho^{2}-\rho^{2}\beta^{2}{\left(\tau -\tau_{0}\right)}^{2}\right)\approx \frac{\exp\left(2\rho^{2}-\rho^{2}\beta^{2}{\left(\tau -\tau_{0}\right)}^{2}\right)}{\sqrt{2\pi \left(2\rho^{2}-\rho^{2}\beta^{2}{\left(\tau -\tau_{0}\right)}^{2}\right)}}\left(1+O\left(\frac{1}{\rho^{2}}\right)\right)$$

In the high-SNR regime, the posterior probability mass is concentrated in a narrow neighborhood around $\tau_{0}$. In this region, the square-root prefactor in (33) varies much more slowly than the exponential term and only introduces a higher-order algebraic correction. Hence, the dominant τ-dependence of the posterior PDF is governed by the exponential kernel. Substituting the leading-order term of (33) into (31) and absorbing all τ-independent factors into the normalization, the posterior PDF can be rewritten as

(34) $$p\left(\tau |z\right)\approx \frac{\exp\left(-\rho^{2}\beta^{2}{\left(\tau -\tau_{0}\right)}^{2}\right)}{\int_{-N/2}^{N/2-1}\exp\left(-\rho^{2}\beta^{2}{\left(\tilde{\tau }-\tau_{0}\right)}^{2}\right)d\tilde{\tau }}$$

Accordingly, the posterior PDF is asymptotically Gaussian:

(35) $$p\left(\tau |z\right)=\frac{1}{\sqrt{2\pi \sigma_{\tau }^{2}}}\exp\left(-\frac{{\left(\tau -\tau_{0}\right)}^{2}}{2\sigma_{\tau }^{2}}\right)$$

The posterior variance is obtained by coefficient matching as

(36) $$\sigma_{\tau }^{2}=\frac{\sigma_{w}^{2}}{2\alpha_{0}^{2}K}=\frac{1}{2\rho^{2}\beta^{2}}$$

This variance is determined by the second-order curvature of the log-posterior around $\tau_{0}$, as induced by the second-order Taylor expansion of the signal autocorrelation function. Since the high-SNR maximum a posteriori (MAP) estimator asymptotically coincides with the matched-filter-based MLE, the same quantity $\sigma_{\tau }^{2}$ also characterizes the asymptotic variance of that estimator.The neglected non-exponential terms only contribute $O\left(1/\rho^{2}\right)$-order corrections to the subsequent entropy and RMI expressions.

C. **Closed-form RMI**

The prior distribution of τ is uniform over [−N/2,N/2), its differential entropy is

(37) $$H\left(\tau \right)=\log_{2}N$$

Since the posterior PDF admits a Gaussian approximation in the high-SNR regime, the conditional differential entropy can be expressed, up to $O\left(1/\rho^{2}\right)$, as

(38) $$H\left(\tau |z\right)=\frac{1}{2}\log_{2}\left(2\pi e\sigma_{\tau }^{2}\right)+O\left(\frac{1}{\rho^{2}}\right)$$

Substituting (38) into (18), the RMI can be written as

(39) $$I\left(\tau ;z\right)=\log_{2}\left(N\right)-\frac{1}{2}\log_{2}\;\left(2\pi e\sigma_{\tau }^{2}\right)+O\left(\frac{1}{\rho^{2}}\right)$$

Substituting $\sigma_{\tau }^{2}=1/2\rho^{2}\beta^{2}$ into (39), we obtain the RMI in sensing-unconstrained scenario:

(40) $$I\left(\tau ;z\right)=\log_{2}\frac{N\beta \rho }{\sqrt{\pi e}}+O\left(\frac{1}{\rho^{2}}\right)$$

Therefore, the closed-form RMI in (40) is directly determined by the Gaussian posterior variance $\sigma_{\tau }^{2}$, which also coincides with the asymptotic local uncertainty of the matched-filter peak estimator in the high-SNR regime.

### 3.2. Constrained Scenario

In practical ISAC systems, the sensing operation is typically confined to a finite detection range due to physical propagation limits, hardware constraints, and system-level configurations.

A. **Posterior PDF**

In the constrained scenario, the sensing interval is explicitly restricted to [a,b]. We define the interval as W=b−a. The prior distribution of the target time delay can be expressed as a uniform distribution over this interval

(41) $$p\left(\tau \right)=\frac{1}{W}$$

Since only the probability mass within [a,b] is retained, the integral of $\tilde{p}\left(\tau |z\right)$ over this interval is generally smaller than 1, and a normalization step is required. To obtain a valid posterior PDF, we define the normalization constant *Z* as

(42) $$Z=\int_{a}^{b}\frac{1}{\sqrt{2\pi \sigma_{\tau }^{2}}}\exp\left(-\frac{{\left(\tau -\tau_{0}\right)}^{2}}{2\sigma_{\tau }^{2}}\right)d\tau$$

which represents the retained probability mass of the Gaussian posterior within the sensing interval [a,b]. Then, the normalized posterior distribution is given by

(43) $$\begin{array}{cc} p(\tau |z) & =\frac{1}{Z}\cdot \frac{1}{\sqrt{2\pi \sigma_{\tau }^{2}}}\exp\left(-\frac{{\left(\tau -\tau_{0}\right)}^{2}}{2\sigma_{\tau }^{2}}\right)\\ \; & =\frac{\exp\left(-\frac{{\left(\tau -\tau_{0}\right)}^{2}}{2\sigma_{\tau }^{2}}\right)}{\int_{a}^{b}\exp\left(-\frac{{\left(\tilde{\tau }-\tau_{0}\right)}^{2}}{2\sigma_{\tau }^{2}}\right)d\tilde{\tau }},\;\tau \in \left[a,b\right] \end{array}$$

Equation (43) shows that the constrained posterior is obtained by normalizing the Gaussian posterior kernel over the finite interval [a,b]. Therefore, the resulting posterior is a truncated Gaussian distribution induced by the sensing constraint. To facilitate the subsequent entropy and MI analysis, the normalization term can be further expressed using the cumulative distribution function (CDF) of a standard Gaussian random variable. The posterior distribution admits the closed-form expression

(44) $$p\left(\tau |z\right)=\frac{\phi \left(u\right)}{\sigma_{\tau }\left[\Phi \left(B\right)-\Phi \left(A\right)\right]}=\frac{\phi \left(u\right)}{\sigma_{\tau }Z},\;\tau \in \left[a,b\right]$$

where $u=\left(\tau -\tau_{0}\right)/\sigma_{\tau }$, $A=\left(a-\tau_{0}\right)/\sigma_{\tau }$, $B=\left(b-\tau_{0}\right)/\sigma_{\tau }$, and ϕ(·) and Φ(·) are the standard normal PDF and CDF, respectively. The normalization term *Z* defined above can be equivalently written as Z=Φ(B)−Φ(A), which denotes the retained posterior probability of the Gaussian approximation over the sensing interval [a,b].

B. **Posterior entropy of truncated Gaussian**

Based on the truncated posterior in (43), which is induced by the high-SNR Gaussian approximation of the unconstrained posterior, the conditional posterior entropy can be written as

(45) $$H\left(\tau |z\right)=-\int_{a}^{b}p\left(\tau |z\right)\log_{2}p\left(\tau |z\right)d\tau$$

Substituting (44) into (45), the posterior entropy of τ given the observation z can be written as

(46) $$\begin{array}{ccc} H\left(\tau |z\right) & = & -\int_{a}^{b}\frac{\phi \left(u\right)}{\sigma_{\tau }Z}\log_{2}\left(\frac{\phi \left(u\right)}{\sigma_{\tau }Z}\right)d\tau \\ & = & -\int_{A}^{B}\frac{\phi \left(u\right)}{Z}\log_{2}\left(\frac{\phi \left(u\right)}{\sigma_{\tau }Z}\right)du\\ & = & -\left[\int_{A}^{B}\frac{\phi \left(u\right)}{Z}\left(-\frac{1}{2\ln2}u^{2}-\frac{1}{2}\log_{2}\left(2\pi \right)\right)du-\log_{2}\left(\sigma_{\tau }Z\right)\int_{A}^{B}\frac{\phi \left(u\right)}{Z}du\right]\\ & = & -\left[-\frac{1}{2Z\ln2}\int_{A}^{B}u^{2}\phi \left(u\right)du-\frac{1}{2}\log_{2}\left(2\pi \right)-\log_{2}\left(\sigma_{\tau }Z\right)\right]\\ & = & \log_{2}\left(\sigma_{\tau }Z\right)+\frac{1}{2}\log_{2}\left(2\pi \right)+\frac{1}{2Z\ln2}\int_{A}^{B}u^{2}\phi \left(u\right)du \end{array}$$

The third term in (46) can be expressed using properties of the standard normal distribution

(47) $$\int_{A}^{B}u^{2}\phi \left(u\right)du=\int_{A}^{B}\phi \left(u\right)du-\left(B\phi (B)-A\phi (A)\right)=Z-\left(B\phi (B)-A\phi (A)\right)$$

which accounts for the second-order moment of the truncated Gaussian. Substituting (47) into (46), the posterior entropy can be expressed as

(48) $$\begin{array}{ccc} H(\tau |z) & = & \log_{2}\left(\sigma_{\tau }Z\right)+\frac{1}{2}\log_{2}\left(2\pi \right)+\frac{1}{2Z\ln2}\left(Z-(B\phi (B)-A\phi (A))\right)\\ & = & \log_{2}\left(\sigma_{\tau }\sqrt{2\pi e}\right)+\log_{2}Z-\frac{B\phi (B)-A\phi (A)}{2Z\ln2}+O\left(\frac{1}{\rho^{2}}\right) \end{array}$$

Since the underlying unconstrained posterior is accurate up to higher-order high-SNR terms, the entropy expression in (48) is also valid up to $O\left(1/\rho^{2}\right)$. The term $\log_{2}\left(Z\right)$ reflects the normalization effect caused by the finite sensing interval, whereas the term $-\left(B\phi (B)-A\phi (A)\right)/\left(2Z\ln2\right)$ is an entropy correction term that quantifies the effect of boundary constraints relative to the unconstrained Gaussian scenario.

C. **Closed-form RMI**

Since the prior distribution of the delay is uniform over $\left[a,b\right]$, its differential entropy is

(49) $$H\left(\tau \right)=\log_{2}\left(b-a\right)=\log_{2}W$$

Therefore, the corresponding RMI approximation in the sensing-constrained scenario is

(50) $$\begin{array}{ccc} I(\tau ;z) & = & H(\tau )-H(\tau |z)\\ & = & \log_{2}W-\left[\left.\log_{2}\left(\sigma_{\tau }\sqrt{2\pi e}\right)+\log_{2}Z-\frac{B\phi (B)-A\phi (A)}{2Z\ln2}\right]\right.\\ & = & \log_{2}W-\log_{2}\left(\sigma_{\tau }\sqrt{2\pi e}\right)-\log_{2}Z+\frac{B\phi (B)-A\phi (A)}{2Z\ln2}\\ & = & \log_{2}\frac{W\beta \rho }{\sqrt{\pi e}Z}+\frac{B\phi (B)-A\phi (A)}{2Z\ln2}+O\left(\frac{1}{\rho^{2}}\right) \end{array}$$

The correction terms in (50) quantify the entropy change caused by the finite sensing interval. Here, $A=\left(a-\tau_{0}\right)/\sigma_{\tau }$ and $B=\left(b-\tau_{0}\right)/\sigma_{\tau }$ are the normalized distances from the posterior mean to the interval boundaries. When $\tau_{0}$ is far from the boundaries, ϕ(A) and ϕ(B) are negligible, so the correction term vanishes and the result reduces to the unconstrained Gaussian case. When $\tau_{0}$ approaches a boundary, the truncation effect becomes significant, and the correction term describes the corresponding entropy reduction.

Substituting $\sigma_{\tau }^{2}=1/2\rho^{2}\beta^{2}$, we obtain the RMI as follows:

(51) $$I\left(\tau ;z\right)=\log_{2}\frac{W\beta \rho }{\sqrt{\pi e}Z}+\frac{B\phi (B)-A\phi (A)}{2Z\ln2}+O\left(\frac{1}{\rho^{2}}\right)$$

We let the sensing interval width be written as

(52) W=λN

where λ is a scaling factor. Substituting it into (51), the RMI in the sensing-constrained scenario is

(53) $$I\left(\tau ;z\right)=\log_{2}\frac{\lambda N\beta \rho }{\sqrt{\pi e}\;Z}+\frac{B\phi (B)-A\phi (A)}{2Z\ln2}+O\left(\frac{1}{\rho^{2}}\right)$$

From the derived expression, the RMI between the delay parameter and the observation is explicitly determined by both system parameters and sensing interval constraints. Specifically, the RMI increases logarithmically with the scaling factor λ, root mean square bandwidth β, and the SNR, indicating that higher bandwidth, higher SNR, and a wider sensing range lead to improved delay information extraction.

### 3.3. MLE for Range Estimation

**Figure 2:**
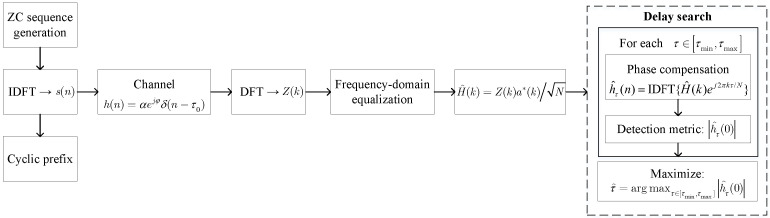
Numerical MLE-based flowchart for target range estimation using ZC sequence and frequency domain matched filtering.

To validate the effectiveness of the proposed closed-form approximations, an MLE-based target range estimation method is adopted for numerical evaluation, and the detailed implementation steps are illustrated in Figure 2.

The transmitted signal is constructed using a Zadoff–Chu (ZC) sequence of length *N*. The sequence is generated in the frequency domain and converted to the time domain using the inverse discrete Fourier transform (IDFT). A cyclic prefix is added to enable circular convolution with the channel impulse response. A single point target is considered, with the channel impulse response given by

(54) $$h\left(n\right)=\alpha e^{j\varphi }\delta \left(n-\tau_{0}\right)$$

where $\tau_{0}$ denotes the real target delay. The received signal is expressed as

(55) z(n)=s(n)∗h(n)+w(n)

At the receiver, the received signal is transformed into the frequency domain. Frequency domain equalization is then applied to estimate the channel frequency response as

(56) $$\hat{H}\left(k\right)=\frac{Z\left(k\right)a^{*}\left(k\right)}{\sqrt{N}}$$

where Z(k) denotes the DFT of the received signal and a(k) is the transmitted ZC sequence. This operation corresponds to a frequency domain matched filtering with respect to the transmitted ZC sequence.

To estimate the target delay, a delay-matched filtering operation is performed over a predefined sensing interval $\left[\tau_{\min},\tau_{\max}\right]$. For each candidate delay τ, phase compensation is applied in the frequency domain, yielding

(57) $${\hat{h}}_{\tau }\left(n\right)=\mathrm{IDFT}\left\{\hat{H}\left(k\right)e^{j2\pi k\tau /N}\right\}$$

When the hypothesized delay τ coincides with the real target delay, the energy of ${\hat{h}}_{\tau }\left(n\right)$ is maximally concentrated at the zero-delay tap. We define the detection metric as $\left|{\hat{h}}_{\tau }\left(0\right)\right|$, which corresponds to the magnitude of the reconstructed channel impulse response at the expected target location. A larger value of $\left|{\hat{h}}_{\tau }\left(0\right)\right|$ indicates a higher likelihood that the real target delay coincides with the tested hypothesis τ.

The target delay estimate is then obtained by maximizing the detection metric over the sensing interval, i.e.,

(58) $$\hat{\tau }=\arg\max_{\tau \in [\tau_{\min},\tau_{\max}]}\left|{\hat{h}}_{\tau }\left(0\right)\right|$$

## 4. Numerical Results and Analysis

In this section, numerical simulations are performed to validate the proposed high-SNR posterior approximations and the resulting closed-form RMI expressions under both sensing-unconstrained and sensing-constrained scenarios. For both scenarios, the posterior PDF of the target range and the corresponding RMI are jointly evaluated. The numerical results are compared with the corresponding theoretical expressions in order to verify the Gaussian and truncated-Gaussian posterior models, as well as the accuracy of the derived RMI approximations under different SNRs, sensing interval widths, sequence lengths, and target positions. In this way, the numerical results not only illustrate the posterior behavior under different sensing conditions, but also provide direct support for the credibility of the developed analytical framework.

Specifically, a ZC sequence is used as the sensing sequence and mapped onto the OFDM subcarriers in the frequency domain. The transmit waveform is generated by an *N*-point IDFT followed by CP insertion, so the simulated signal is an OFDM waveform consistent with the MLE-based signal model. A single-point target is considered. The main simulation parameters are set as follows: N=16, ZC root r=1, cyclic shift q=0, CP length N/2=8, target amplitude α=1, and random phase $\varphi \in \left[0,2\pi \right]$. The target delay is fixed at the center of the observation interval.

**Figure 3:**
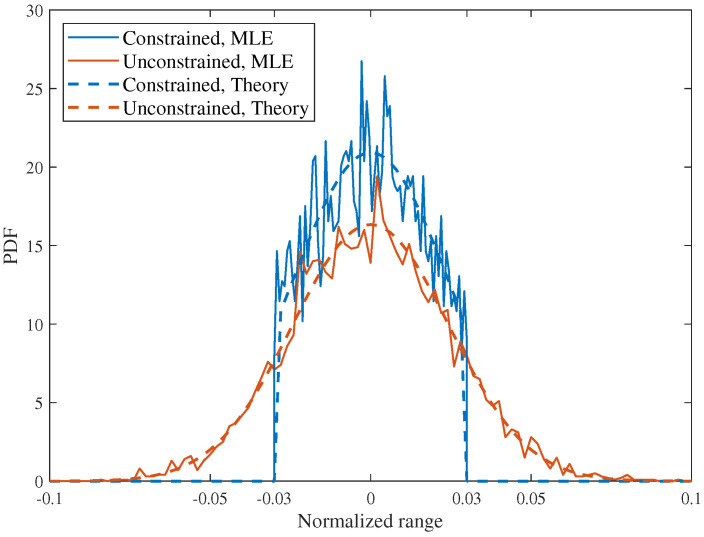
Comparison of PDF versus normalized range in sensing-constrained and sensing-unconstrained scenarios, SNR = 15 dB, N=16.

Figure 3 compares the posterior PDFs in the sensing-unconstrained and sensing-constrained scenarios for N=16 at SNR = 15 dB. In the constrained scenario, the sensing interval is set to $\left[-0.03,0.03\right]$, whereas in the unconstrained scenario no finite support is imposed. It is observed that the MLE-based numerical results agree with the corresponding theoretical curves in both scenarios, which validates the derived closed-form posterior expressions. For the unconstrained scenario, the posterior exhibits the Gaussian-like shape predicted by the high-SNR analysis. After introducing the finite sensing interval, the posterior is truncated within the feasible region and becomes more concentrated around the true target position. In particular, the peak value in the constrained scenario is about 40% higher than that in the unconstrained scenario, and the PDF near the interval edges is also visibly lifted due to boundary restrictions. This indicates that the constrained sensing interval does not alter the intrinsic high-SNR concentration behavior of the posterior but reshapes its support and increases its central concentration.

**Figure 4:**
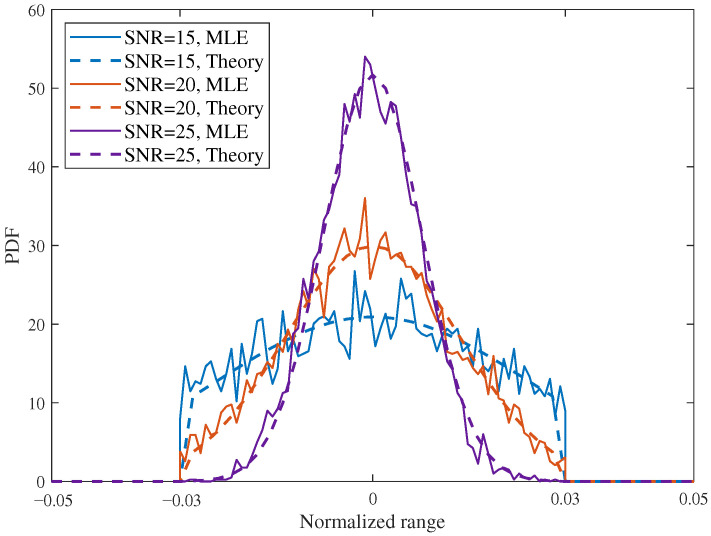
Comparison of PDF in sensing-constrained scenario with different SNR, SNR = 15, 20, 25 dB, N=16.

Figure 4 shows the posterior PDFs in the sensing-constrained scenario for N=16 and sensing interval $\left[-0.03,0.03\right]$ under different SNRs, namely 15 dB, 20 dB, and 25 dB. The theoretical curves remain in close agreement with the MLE-based numerical results over all SNR values, confirming that the proposed closed-form posterior approximation accurately captures the SNR-dependent posterior results. As the SNR increases from 15 dB to 20 dB and then to 25 dB, the posterior becomes progressively sharper and more concentrated around the true target position. Compared with the 15 dB case, the peak value at 20 dB increases by about 45%, while at 25 dB it becomes more than twice the level at 15 dB. At the same time, the main lobe width shrinks noticeably, which means that the posterior uncertainty is substantially reduced as the sensing reliability improves. These results are fully consistent with the theoretical analysis, and they also show that the MLE-based numerical evaluation can effectively verify the accuracy of the derived closed-form expression over different SNR regimes.

**Figure 5:**
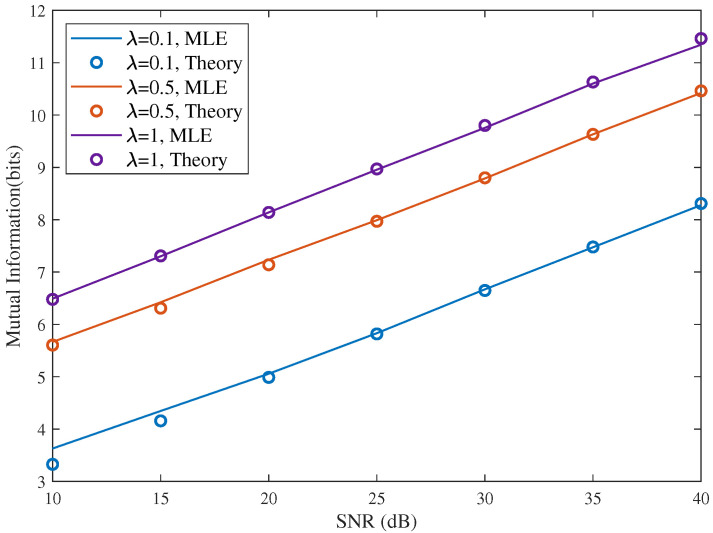
Comparison of RMI with different sensing interval *W*, λ=0.1,0.5,1 is the ratio of sensing interval width to the full interval W=λN, N=16.

Figure 5 presents the RMI versus SNR for different sensing interval scaling factors, where the interval width is defined as W=λN and the sequence length is fixed. The results are shown for λ=0.1, λ=0.5, and λ=1. It can be seen that the theoretical results almost overlap with the MLE-based numerical curves over the whole SNR range, which directly validates the proposed closed-form RMI expression. For all three values of λ, the RMI increases monotonically with SNR, while a larger sensing interval leads to a higher mutual information. Quantitatively, enlarging the interval from λ=0.1 to λ=1 yields an RMI increase of about 2.8 bits throughout the range, indicating that a narrow sensing window imposes a strong limitation on the achievable sensing information. By contrast, the gap between λ=0.5 and λ=1 is much smaller, generally below 1 bit, which means that once the interval width reaches a moderate level, further enlargement provides only a limited additional gain.

**Figure 6:**
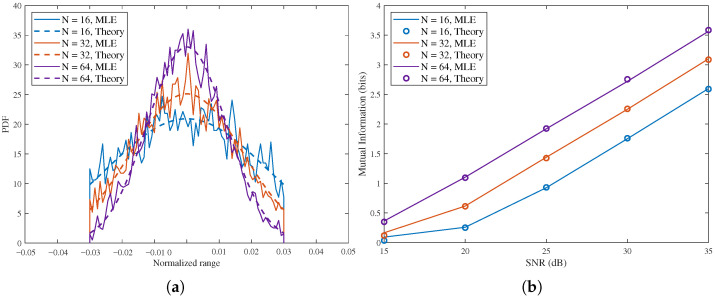
PDF and RMI in sensing-constrained scenario with different *N*, N=16,32,64. (**a**) Posterior PDF, SNR = 15 dB. (**b**) RMI versus SNR.

Figure 6 shows the PDF and RMI in sensing-constrained scenario with different *N*. In Figure 6a, with SNR = 15 dB and N=16,32,64, the posterior becomes progressively sharper as *N* increases, the peak rises from about 21 for N=16 to about 27 for N=32, and further to about 35 for N=64, while the spread around the true target position is clearly reduced. This means that a larger *N* yields a more concentrated posterior and a smaller estimation uncertainty. The reason is that a longer sequence provides more effective observations and stronger delay discrimination, so the likelihood around the true delay becomes steeper and the posterior mass is compressed into a narrower region. The same effect appears in Figure 6b, where the RMI increases with both SNR and *N*. For example, at 25 dB, the RMI is about 0.9 bit, 1.4 bits, and 1.9 bits for N=16,32,64, respectively; at 35 dB, these values increase to about 2.6 bits, 3.1 bits, and 3.6 bits. Therefore, increasing *N* improves sensing performance in a consistent way. It sharpens the posterior PDF and at the same time increases the achievable mutual information. The theoretical and MLE-based numerical curves remain closely matched in both subfigures, which shows that the derived closed-form expressions correctly capture the impact of sequence length.

**Figure 7:**
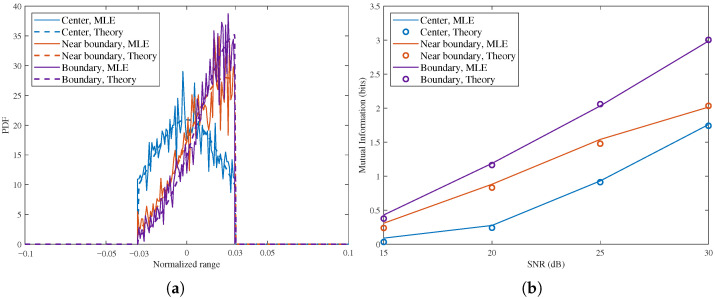
PDF and RMI in sensing-constrained scenario with different target position, the target in the center, near the boundary and in the boundary, N=16. (**a**) Posterior PDF, SNR = 15 dB. (**b**) RMI versus SNR.

Figure 7 shows the effect of target position on the posterior PDF and the corresponding RMI in the sensing-constrained scenario for N=16. In Figure 7a, with SNR = 15 dB, the posterior is nearly symmetric when the target is at the center of the sensing interval and becomes increasingly skewed as the target moves toward the near-boundary and boundary positions. Meanwhile, the peak increases from about 22 at the center to about 30 near the boundary and to about 36 at the boundary. This indicates that the posterior becomes more concentrated near the interval edge because the feasible uncertainty region is more strongly truncated there. The same trend appears in Figure 7b. At 30 dB, the RMI is about 1.75 bits, 2.05 bits, and 3.0 bits, respectively, so the boundary case is about 1.25 bits higher than the center case. Therefore, in the constrained scenario, moving the target toward the boundary strengthens the truncation effect, reduces posterior uncertainty, and increases the achievable RMI.

**Figure 8:**
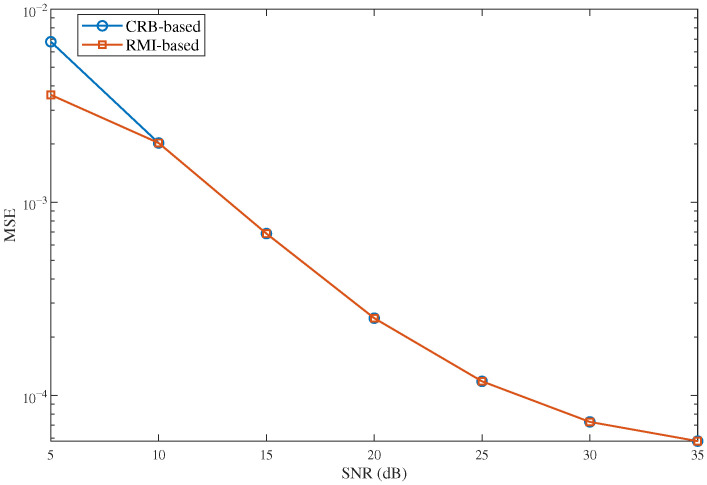
MSE comparison between CRB-based design and RMI-based design under different SNR.

To further discuss the practical implication of the proposed method, Figure 8 presents an MSE comparison between an RMI-based design and a conventional CRB-based design in a boundary-sensitive constrained sensing scenario. The results show that the RMI-based design achieves a visible performance gain in the low-SNR regime, where the posterior PDF is still influenced by finite-support and non-Gaussian effects. As the SNR increases, the two theme tend to be the same, which is consistent with the high-SNR analysis in this paper: once the posterior becomes sharply concentrated, the CRB-based and RMI-based design criteria tend to yield similar estimation performance. However, the main practical value of the proposed method in the high-SNR regime lies in its closed-form and low-complexity RMI characterization. Unlike the CRB, which only quantifies a local error bound, the proposed RMI framework captures the overall posterior uncertainty and explicitly incorporates the effect of finite sensing intervals, making it more suitable for sensing-oriented analysis and design under constrained scenarios.

We further analyze the computational complexity of the proposed analytical evaluation and compare it with a representative grid-search-based numerical baseline. Since many related posterior/RMI evaluation methods rely on numerical search or numerical integration rather than explicit closed-form expressions, the MLE-based grid-search numerical evaluation is adopted here as a practical benchmark for comparison. Under a fixed delay-search resolution, the number of delay hypotheses grows linearly with the signal length *N*. For each delay hypothesis, the MLE-based numerical method requires one frequency domain phase compensation and one *N*-point IFFT. Therefore, the overall computational complexity scales as $O(N^{2}\log N)$. For example, when N=16, the fixed delay-search grid contains approximately 8000 hypotheses. By contrast, once the posterior variance and the boundary-related terms are determined, the proposed closed-form RMI expression can be evaluated directly by elementary operations together with Gaussian PDF/CDF calculations, resulting in a constant computational cost of O(1) for each parameter setting. Therefore, compared with grid-search-based numerical evaluation methods, the proposed analytical approach is substantially more efficient and is more suitable for fast parameter sweeps and repeated performance evaluation.

## 5. Discussion

The results of this paper provide an information-theoretic interpretation of range sensing performance in ISAC systems under sensing-constrained and sensing-unconstrained scenarios. Unlike most existing studies that assume an unbounded sensing interval, the proposed model explicitly accounts for finite detection ranges, which are common in real systems. By modeling the target range posterior PDF, the impact of boundary effects on sensing uncertainty is clearly revealed. The numerical results show that, in the high-SNR regime, the posterior PDF approximately follows a Gaussian form in the unconstrained scenario and a truncated Gaussian in the constrained scenario. The interval constraint does not alter the intrinsic statistical structure of the posterior but restricts the admissible delay support, leading to probability mass redistribution and limited entropy reduction. As a result, although the posterior PDF becomes more concentrated locally, the achievable RMI is fundamentally bounded by the sensing interval. Numerical results further indicate that the impact of interval constraints diminishes as the detection range expands, while SNR and sequence length consistently dominate performance improvement.

Several directions can be explored in future work. First, the proposed framework can be extended to multi-target scenarios, where target interactions and data association may further influence the posterior distribution and RMI. Second, joint estimation of multiple sensing parameters, such as range and velocity, can be investigated to characterize the coupled information gain in range–Doppler ISAC systems. Third, the closed-form RMI expression can be incorporated into waveform and resource optimization problems, enabling adaptive design of sensing intervals, power allocation, and sequence lengths under practical constraints. In addition, extending the analysis to low-SNR regimes, model mismatch, and non-Gaussian noise environments would further enhance the robustness and applicability of the proposed approach in realistic ISAC deployments.

## 6. Conclusions

This paper proposed a novel closed-form approximation of RMI applicable to both constrained and unconstrained scenarios utilizing information theory. We developed an RMI extraction model that effectively captures the fundamental range-dependent characteristics of ISAC systems. To this end, we derived an explicit expression for the PDF of the target range. Theoretical analysis revealed that, under high SNR, the estimated range PDF approximated a Gaussian distribution in the sensing-unconstrained scenario and a truncated Gaussian distribution in the sensing-constrained scenario. These findings underscore the necessity of incorporating the range dimension in sensing performance evaluation. Moreover, we derived closed-form approximations for RMI for both scenarios under high SNR. The proposed approximations showed excellent agreement with numerical results. Performance evaluation revealed that boundary constraints significantly reduce the RMI. Future work could explore dynamic range control strategies to fully exploit the potential of RMI for ISAC systems.

## Figures and Tables

**Table 1:** Comparison of representative related works and the present work.

References	Category	Main Characteristic/Limitation
[11,12,13,14,15,16,17,18]	MI-based ISAC	General MI formulations; no explicit RMI for range sensing
[19,20,21,22,23,24]	Amplitude/phase design	Beamforming/waveform/RIS design; mainly amplitude/phase-oriented
[25,26,27,28,29,30]	Range/posterior analysis	Range estimation/posterior modeling; no closed-form RMI under finite interval
This work	RMI-based range sensing	Explicit range posterior PDF and closed-form high-SNR RMI for constrained/unconstrained sensing

## Data Availability

The raw data supporting the conclusions of this article will be made available by the authors on request.

## Appendix A

**Proof of** (5)**.** According to the signal model,

(A1) $$z=\alpha e^{j\varphi }U\left(\tau \right)s+w$$

where $w∼\mathrm{CN}(0,\sigma_{w}^{2}I_{N})$. Since $\alpha e^{j\varphi }U\left(\tau \right)s$ is deterministic for given (τ,α,φ), it follows that

(A2) $$z\;|\;\left(\tau ,\alpha ,\varphi \right)∼\mathrm{CN}\;\left(\alpha e^{j\varphi }U\left(\tau \right)s,\sigma_{w}^{2}I_{N}\right)$$

Using the standard PDF of an *N*-dimensional complex Gaussian vector,

(A3) $$p\left(x\right)=\frac{1}{\pi^{N}\det\left(C\right)}\exp\;\left[-{\left(x-\mu \right)}^{H}C^{-1}\left(x-\mu \right)\right]$$

and substituting $\mu =\alpha e^{j\varphi }U\left(\tau \right)s$ and $C=\sigma_{w}^{2}I_{N}$, we directly obtain

(A4) $$p\left(z|\tau ,\alpha ,\varphi \right)=\frac{1}{{\left(\pi \sigma_{w}^{2}\right)}^{N}}\exp\left(-\frac{1}{\sigma_{w}^{2}}{∥z-\alpha e^{j\varphi }U\left(\tau \right)s∥}^{2}\right)$$

which is exactly (5). □
